# Addenbrooke's Hospital, Cambridge

**Published:** 1902-05-24

**Authors:** 


### ADDENBROOKE'S HOSPITAL, CAMBRIDGE.

At a special general court of the Governors held at the hospital on the 15th instant, the report of the Advisory Council and the Select Governor, appointed on February 19th to prepare a scheme for submission to the Charity Commissioners, was presented. The Advisory Council recommended the adoption of the scheme which was published in The Hospital on page 347 of the issue of February 15th, 1902, with two alterations providing for an increase in the number of the proposed general committee from 18 to 24, and the retirement of one-half instead of one-third of the members of that committee annually. The special general court, after full discussion, adopted the scheme as amended. As soon, therefore, as the necessary formalities are completed, the government of Addenbrooke's Hospital will be placed in the hands of a general committee, in accordance with the original recommendation of Sir Henry Burdett, which has been under discussion by the Governors since the date of his report, viz., June 1898.

